# Characterization of bovine *MHC DRB3* diversity in global cattle breeds, with a focus on cattle in Myanmar

**DOI:** 10.1186/s12863-020-00905-8

**Published:** 2020-09-01

**Authors:** Guillermo Giovambattista, Kyaw Kyaw Moe, Meripet Polat, Liushiqi Borjigin, Si Thu Hein, Hla Hla Moe, Shin-Nosuke Takeshima, Yoko Aida

**Affiliations:** 1 Nakamura Laboratory, Baton Zone Program, RIKEN Cluster for Science, Technology and Innovation Hub, 2-1 Hirosawa, Wako, Saitama 351-0198 Japan; 2 IGEVET (UNLP-CONICET LA PLATA), Facultad de Ciencias Veterinarias UNLP, B1900AVW, CC 296 La Plata, Argentina; 3 Department of Pathology and Microbiology, University of Veterinary Science, Yezin, Nay Pyi Taw, 05282 Myanmar; 4 Department of Anatomy, University of Veterinary Science, Yezin, Nay Pyi Taw, 05282 Myanmar; 5 Department of Genetics and Animal Breeding, University of Veterinary Science, Yezin, Nay Pyi Taw, 05282 Myanmar; 6 Department of Food and Nutrition, Faculty of Human Life, Jumonji University, 2-1-28 Sugasawa, Niiza-shi, Saitama 352-8510 Japan

**Keywords:** Myanmar cattle, *BoLA-DRB3*, Immunogenomics, Genotyping, Breed diversity, Breed/population identification

## Abstract

**Background:**

Myanmar cattle populations predominantly consist of native cattle breeds (Pyer Sein and Shwe), characterized by their geographical location and coat color, and the Holstein-Friesian crossbreed, which is highly adapted to the harsh tropical climates of this region. Here, we analyzed the diversity and genetic structure of the *BoLA-DRB3* gene, a genetic locus that has been linked to the immune response, in Myanmar cattle populations.

**Methods:**

Blood samples (*n* = 294) were taken from two native breeds (Pyer Sein, *n* = 163 and Shwe Ni, *n* = 69) and a cattle crossbreed (Holstein-Friesian, *n* = 62) distributed across six regions of Myanmar (Bago, *n* = 38; Sagaing, *n* = 77; Mandalay, *n* = 46; Magway, *n* = 46; Kayin, *n* = 43; Yangon, *n* = 44). In addition, a database that included 2428 *BoLA-DRB3* genotypes from European (Angus, Hereford, Holstein, Shorthorn, Overo Negro, Overo Colorado, and Jersey), Zebuine (Nellore, Brahman and Gir), Asian Native from Japan and Philippine and Latin-American Creole breeds was also included. Furthermore, the information from the IPD–MHC database was also used in the present analysis. DNA was genotyped using the sequence-based typing method. DNA electropherograms were analyzed using the Assign 400ATF software.

**Results:**

We detected 71 distinct alleles, including three new variants for the *BoLA-DRB3* gene. Venn analysis showed that 11 of these alleles were only detected in Myanmar native breeds and 26 were only shared with Asian native and/or Zebu groups. The number of alleles ranged from 33 in Holstein-Friesians to 58 in Pyer Seins, and the observed versus unbiased expected heterozygosity were higher than 0.84 in all the three the populations analyzed. The F_ST_ analysis showed a low level of genetic differentiation between the two Myanmar native breeds (F_ST_ = 0.003), and between these native breeds and the Holstein-Friesians (F_ST_ <  0.021). The average *F*_ST_ value for all the Myanmar Holstein-Friesian crossbred and Myanmar native populations was 0.0136 and 0.0121, respectively. Principal component analysis (PCA) and tree analysis showed that Myanmar native populations grouped in a narrow cluster that diverged clearly from the Holstein-Friesian populations. Furthermore, the *BoLA-DRB3* allele frequencies suggested that while some Myanmar native populations from Bago, Mandalay and Yangon regions were more closely related to Zebu breeds (Gir and Brahman), populations from Kayin, Magway and Sagaing regions were more related to the Philippines native breeds. On the contrary, PCA showed that the Holstein-Friesian populations demonstrated a high degree of dispersion, which is likely the result of the different degrees of native admixture in these populations.

**Conclusion:**

This study is the first to report the genetic diversity of the *BoLA-DRB3* gene in two native breeds and one exotic cattle crossbreed from Myanmar. The results obtained contribute to our understanding of the genetic diversity and distribution of *BoLA-DRB3* gene alleles in Myanmar, and increases our knowledge of the worldwide variability of cattle *BoLA-DRB3* genes, an important locus for immune response and protection against pathogens.

## Background

Myanmar, located in tropical South East Asia, is divided into seven topographic regions: Northern Hills, Western Hills, Shan Plateau, Central Belt, Lower Myanmar Delta, Rakhine Coastal Region and Tanintharyi Coastal Strip. The country has a tropical monsoon climate with three seasons: hot (mid-February to mid-May), rainy (mid-May to mid-October) and cool season (mid-October to mid-February).

Myanmar’s livestock population consists of about 18.7 million head of cattle which are distributed throughout the country, but most concentrated in the central region [1]. Currently, the Myanmar cattle population can be separated into the two native breeds (Shwe Ni and Pyer Sein) and the Holstein-Friesian crossbreed. These populations are predominantly made up of animals with a low degree of artificial selection but which are adapted to the harsh tropical environment, resistant to tropical diseases and external parasites, and able to thrive on low-quality roughages and grasses [2]. Myanmar native cattle are zebu type (*Bos indicus*) cattle and are characterized by their geographical location and coat color. The indigenous breeds Shwe Ni and Pyer Sein are mainly used for draught and to a lesser extent dairy production [3]. Only older animals who are no longer fit for work are slaughtered for beef consumption.

Pyer Sein breed was obtained by crossing Indian and native cattle breeds. Indian breeds included Red Shindi, Hariarna and Thari imported during British colonial times and after Myanmar independence to improve the local milk production. Shwe Ni, the true indigenous Myanmar cattle are mostly red or pied and moderately small, compact and with fine bones [3]. In addition, European dairy breeds including Friesians, Jersey, Guernsey and Norwegian Reds were brought into the country in the second half of the twentieth century. Nowadays, the Holstein-Friesian crossbreed is the most popular dairy cow in Myanmar.

The major histocompatibility complex (MHC) is a major component of the adaptive immune system; with MHC genes encoding the cell-surface glycoproteins that bind small peptide fragments derived from host- and pathogen-expressed proteins via proteolysis [4]. The bovine MHC, known as the bovine leukocyte antigen (BoLA), includes three copies of the *DRB class II* gene with the *BoLA-DRB3* variant being the most highly expressed and polymorphic [5]. This locus’ polymorphisms influence both the magnitude and epitope specificity of the antigen-specific T cell response to infectious diseases [6]. Polymorphic sites within the *BoLA-DRB3* gene are mainly found at the peptide-binding sites that comprise the α1 and β1 domains of the protein, with most maintained by balancing or overdominance selection [7, 8]. The *BoLA-DRB3* gene is associated with resistance/susceptibility to infectious disease (e.g., bovine leukosis virus-induced lymphocytosis, mastitis and dermatophilosis), different immunological and production traits (e.g., milk yield) and different vaccine responses (e.g., to foot-and-mouth disease and Theileria parva) [9, 10]. For these reasons, the study of the genetic variability in bovine MHC genes is of interest for evolutionary biologists and animal sciences researchers, as well as, veterinarians, breeders and cattle owners. The analysis of the genetic diversity of the *BoLA-DRB3* gene began more than 25 years ago with pioneering studies using serotype analysis [11, 12]. Later, different genotyping techniques were applied (sequencing of cloned genomic DNA, cDNA, cloned PCR products or PCR-RFLP) to several different bovine breeds (e.g., [13–15]). Currently, polymerase chain reaction-sequence based typing (PCR-SBT) [16–27] and target next generation sequencing (Target-NGS) [28] are the most commonly employed and powerful tools used in this type of analysis. However, these techniques have been applied in studies which have included only a handful of the over 800 recognized cattle breeds worldwide [16–28]. Consequently, there are still a number of breeds that remain uncharacterized, and this number increases when local native bovine breeds are considered [17, 18, 26, 29]. As a result of these studies there are 144 described *BoLA-DRB3* alleles for bovines, and 303 subtypes listed in the Immuno Polymorphism Database (IPD-MHC) [30, 31]. In addition, previous studies [16–27] have shown an even distribution of *BoLA-DRB3* polymorphisms between the major bovine types (*Bos taurus* and *B. indicus*) and breeds. This is likely as a result of various factors including breed origin, artificial or natural selection and geographical distribution.

The aim of this work was to assess, at both the allele and molecular levels, the inherent genetic diversity and structure of the *BoLA-DRB3* gene in Myanmar cattle.

## Results

### Distribution of *BoLA-DRB3* alleles between selected Myanmar cattle breeds

**Fig. 1:**
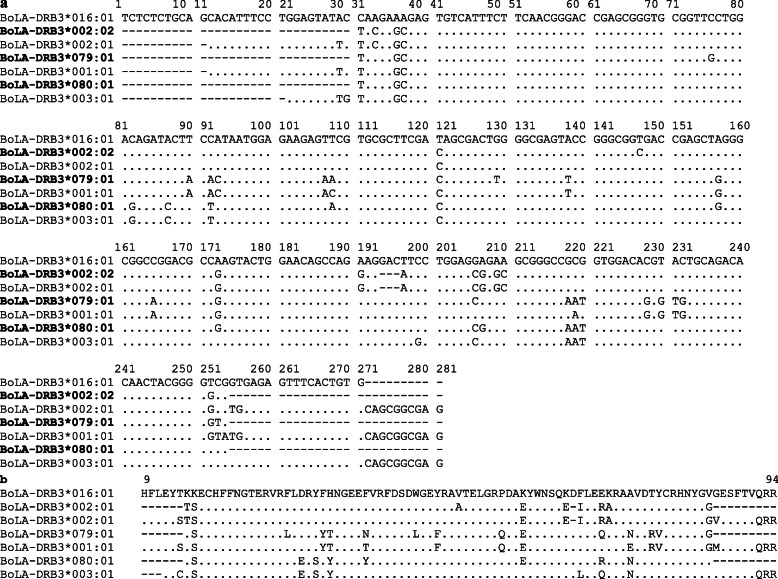
Alignment of the nucleotide (**a**) and the predicted amino acid (**b**) sequences of the β1 domain encoded by three new *BoLA-DRB3* alleles (accession numbers BoLA09870 for BoLA-DRB3*002:02, BoLA09869 for BoLA-DRB3*079:01, and BoLA09871 for BoLA-DRB3*080:01) derived from 294 cattle (163 animals of the Myanmar Pyer Sein native, 69 Myanmar Shwe Ni native cattle breeds, and 62 animals of the Holstein-Friesian crossbreed). Numbering refers to amino acid positions in the mature protein. New alleles are indicated in bold. Nucleotide and amino acid residues identical to those encoded by the *BoLA-DRB3* cDNA clone NR-1 are indicated by dots (correspond to GenBank accession number D45357 and the allele BoLA-DRB3*016:01; Aida et al., 1995). The most similar BoLA-DRB3 alleles (BoLA-DRB3*001:01, BoLA-DRB3*003:01 and BoLA-DRB3*002:01) were aligned below each new variant. Missing data are indicated by dashes. Closer *BoLA-DRB3* alleles with new variants are also included in the figure

PCR-SBT genotyping allowed us to identify 72 *BoLA-DRB3* alleles (69 previously reported variants and three new alleles; Table 1) for the breeds selected in this study. The number of alleles (n_a_) was 57 in Pyer Sein cattle (54 previously reported and three new), 43 in Shwe Ni (41 previously reported and two new), and 33 in the Holstein-Friesian crossbreed (32 previously reported and one new) (Tables 1 and 2). Nucleotide and predicted amino acid sequences of the three new allele variants are shown in Fig. 1.

**Table 1:** BoLA-DRB3 allele frequencies in Myanmar cattle breeds

***BoLA-DRB3*** alleles	Pyer Sein	Shwe Ni	Holstein-Friesian Crossbreed
	(***N*** = 163)	(***N*** = 69)	(***N*** = 62)
BoLA-DRB3*001:01	1.53	1.45	**6.45**
BoLA-DRB3*002:01	3.68	**6.52**	**6.45**
BoLA-DRB3*002:02	**0.31**	0.00	0.00
BoLA-DRB3*003:01	0.31	0.00	0.00
BoLA-DRB3*003:02:01	1.23	0.00	0.00
BoLA-DRB3*005:01	0.31	0.00	4.84
BoLA-DRB3*005:02	0.31	0.00	0.00
BoLA-DRB3*005:03	0.00	0.72	0.00
BoLA-DRB3*005:04	0.00	0.00	0.81
BoLA-DRB3*006:01	0.00	0.00	0.81
BoLA-DRB3*007:01	2.15	2.17	0.81
BoLA-DRB3*008:01	0.00	0.00	1.61
BoLA-DRB3*009:01	4.29	0.72	0.81
BoLA-DRB3*009:02	1.84	2.90	4.84
BoLA-DRB3*010:01	2.76	3.62	4.84
BoLA-DRB3*011:01	0.00	0.00	**11.29**
BoLA-DRB3*011:03	0.92	2.90	0.00
BoLA-DRB3*011:04	0.31	0.00	0.00
BoLA-DRB3*012:01	1.53	1.45	**11.29**
BoLA-DRB3*013:01	1.53	2.17	1.61
BoLA-DRB3*014:01:01	3.68	1.45	4.03
BoLA-DRB3*013:02	0.61	0.00	0.00
BoLA-DRB3*013:03	0.00	0.72	0.00
BoLA-DRB3*015:01	2.76	1.45	3.23
BoLA-DRB3*016:01	2.15	2.17	2.42
BoLA-DRB3*016:02	0.31	0.00	0.00
BoLA-DRB3*017:01	0.00	0.00	3.23
BoLA-DRB3*017:03	**6.13**	**5.07**	0.00
BoLA-DRB3*018:01	1.53	**7.97**	0.00
BoLA-DRB3*01901	2.15	1.45	1.61
BoLA-DRB3*020:01:02	0.00	0.72	**5.65**
BoLA-DRB3*020:02	0.31	0.00	0.00
BoLA-DRB3*20:03	0.61	0.00	0.00
BoLA-DRB3*20:05	0.00	0.72	0.00
BoLA-DRB3*022:01	**6.13**	**5.07**	2.42
BoLA-DRB3*025:01:01	2.45	0.72	1.61
BoLA-DRB3*025:01:02	0.31	0.72	0.00
BoLA-DRB3*025:02	0.00	0.00	0.81
BoLA-DRB3*026:01	4.60	**5.80**	3.23
BoLA-DRB3*027:03	2.45	2.90	0.00
BoLA-DRB3*027:05	0.00	0.00	0.00
BoLA-DRB3*027:07	0.31	2.17	0.00
BoLA-DRB3*027:10	0.61	0.00	0.00
BoLA-DRB3*028:01	3.68	2.90	0.00
BoLA-DRB3*028:02	0.61	0.72	1.61
BoLA-DRB3*030:01	1.84	2.17	0.81
BoLA-DRB3*31:01	**6.75**	1.45	3.23
BoLA-DRB3*31:03	0.31	0.00	0.00
BoLA-DRB3*033:01	0.61	0.72	0.81
BoLA-DRB3*34:01	2.45	0.00	0.00
BoLA-DRB3*034:03	0.31	0.00	0.00
BoLA-DRB3*035:01	1.53	2.17	0.00
BoLA-DRB3*036:01	1.23	0.72	0.00
BoLA-DRB3*037:01	0.61	0.00	0.00
BoLA-DRB3*038:01	0.92	0.00	0.81
BoLA-DRB3*039:01	1.84	0.00	0.00
BoLA-DRB3*041:01	1.53	2.90	0.00
BoLA-DRB3*042:01	0.61	0.00	1.61
BoLA-DRB3*043:01	3.37	1.45	4.03
BoLA-DRB3*043:02	0.00	0.00	0.81
BoLA-DRB3*048:02	1.53	**5.07**	0.00
BoLA-DRB3*049:01	0.00	0.72	0.00
BoLA-DRB3*50:01	0.31	0.00	0.00
BoLA-DRB3*50:11	0.00	0.72	0.00
BoLA-DRB3*57:02	0.61	2.17	0.00
BoLA-DRB3*58:01	0.92	0.00	0.00
BoLA-DRB3*63:01	0.00	0.72	0.00
BoLA-DRB3*64:02	0.61	0.00	0.00
BoLA-DRB3*72:01	1.53	3.62	0.00
BoLA-DRB3*73:01	1.53	2.17	0.81
BoLA-DRB3*079:01	**3.68**	**4.35**	**0.81**
BoLA-DRB3*080:01	**0.61**	**1.45**	0.00

^a^N, number of animals analyzed; ^b^Frequent alleles in each breed are indicated in bold (> 5%); ^c^Novel alleles identified in this study are indicated in bold and underlined

**Table 2:** Sample size **(**N), number of alleles (n_a_), observed (h_o_) and expected (h_e_) heterozygosity, Hardy Weinberg equilibrium (HWE) measured through F_IS_ and Slatkin’s exact test in the cattle breeds studied. F_IS_ *p*-values are indicated between parentheses. Significant *p* values are indicated in bold

Breed	N	n_a_	h_o_	h_e_	HWE	Slatkin’s *p* value
					F_IS_ (*p* value)	
Pyer Sein^a^	163	58	0.94	0.97	0.0276 (0.0534)	**0.006**
Shwe Ni^a^	69	43	0.86	0.97	**0.1197 (< 0.0001)**	**0.010**
Holstein-Friesian crossbreed^a^	62	33	0.94	0.95	0.0183 (0.7794)	0.149
Yacumeño Creole ^b^	112	35	0.92	0.95	0.0344 (0.7363)	**0.001**
Hartón del Valle Creole ^b^	66	24	0.97	0.94	**−0.0360 (< 0.0001)**	0.138
Bolivian Nelore ^c^	116	26	0.78	0.87	0.0990 (0.6921)	0.306
Bolivian Gir ^c^	110	19	0.88	0.92	0.0406 (0.0926)	**0.009**
Nellore x Brahman ^c^	195	33	0.76	0.86	**0.1131 (0.1985)**	0.473
Japanese Holstein ^b^	102	18	0.92	0.90	−0.0215 (0.4481)	0.091
Japanese Shorthorn ^b^	100	20	0.92	0.91	−0.0086 (0.0692)	0.069
Japanese Jersey ^b^	69	14	0.91	0.89	**−0.0297 (0.0023)**	0.042
Japanese Black ^b^	200	23	0.91	0.91	0.0095 (0.4043)	**0.004**
Chilean Hereford ^e^	49	15	0.82	0.87	0.0574 (0.4980)	0.582
Chilean Black Angus ^e^	100	26	0.61	0.90	**0.3246 (< 0.0001)**	0.447
Chilean Red Angus ^e^	99	29	0.71	0.93	**0.2415 (< 0.0001)**	0.080
Philippine Native ^d^	482	71	0.91	0.96	**0.0480 (< 0.0001)**	0.068
Philippine Brahman ^d^	236	58	0.89	0.95	**0.0687 (< 0.0001)**	0.134

^a^Present work; ^b^Giovambattista et al., [26]; ^c^Takeshima et al., [29]; ^d^Takeshima et al., [18]; ^e^Takeshima et al., [22, 23]; and ^f^Takeshima et al., [21]

The three new variants were assigned allele names by IPD-MHC, namely, BoLA-DRB3*002:02, which differs from BoLA-DRB3*002:01 at 147 positions; BoLA-DRB3*079:01, which differs from DRB3*001:01 at seven positions (76, 108, 129, 206, 218, 220 and 254); and BoLA-DRB3*080:01, which differs from BoLA-DRB3*003:01 at five positions (108, 157, 173, 199 and 207). All three new *BoLA-DRB3* allele variants shared about 90–94% and 83.7–90.7% nucleotide and amino acid similarity with the *BoLA-DRB3* cDNA clone NR1 (correspond to GenBank accession number D45357 and the allele BoLA-DRB3*016:01), respectively [5].

**Fig. 2:**
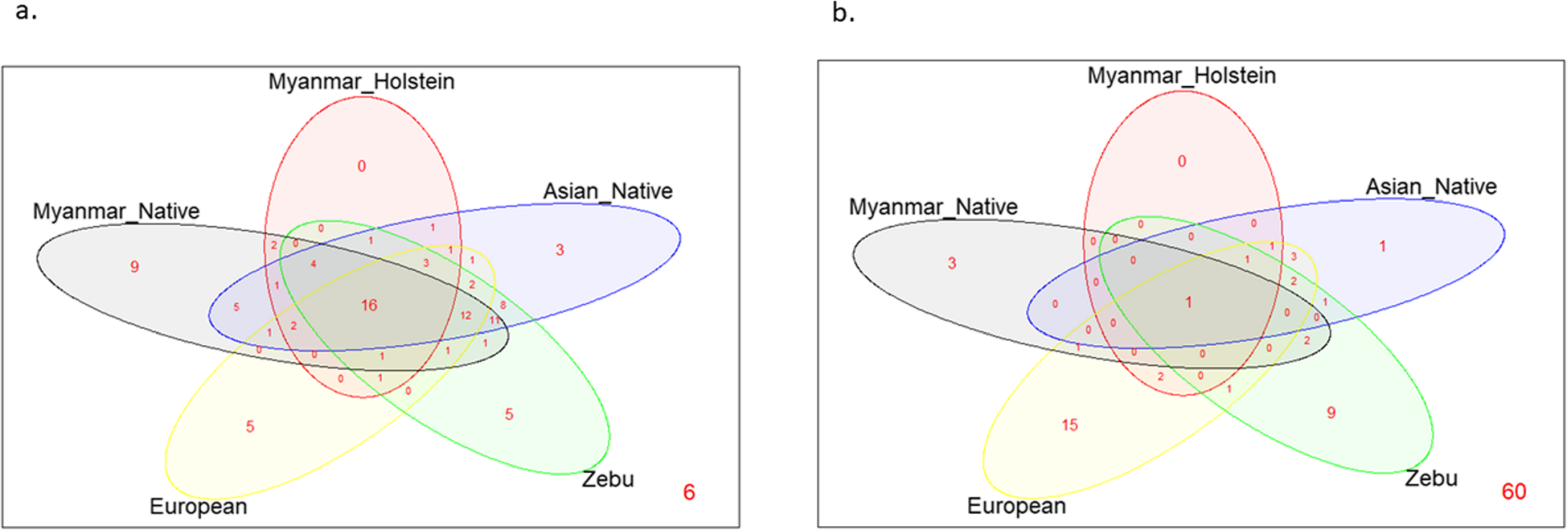
Venn plot of *BoLA-DRB3* alleles shared by Myanmar native breeds (Pyer Sein and Shwe Ni) and Holstein-Friesian crossbreed, Asian native (Philippine native and Japanese Black), Zebu (Bolivian Nellore, Bolivian Gir, Peruvian Nellore-Brahman, and Philippine Brahman), and European (Chilean Hereford, Chilean Black Angus, Chilean Red Angus, Japanese Jersey, Japanese Shorthorn, and Japanese Holstein) breeds. **a** All alleles were considered and b. only alleles which exhibited gene frequencies that were higher than 0.5% were counted. Values at the bottom right of the figures are referring to alleles absent in all group analyzed but present in our database

**Fig. 3:**
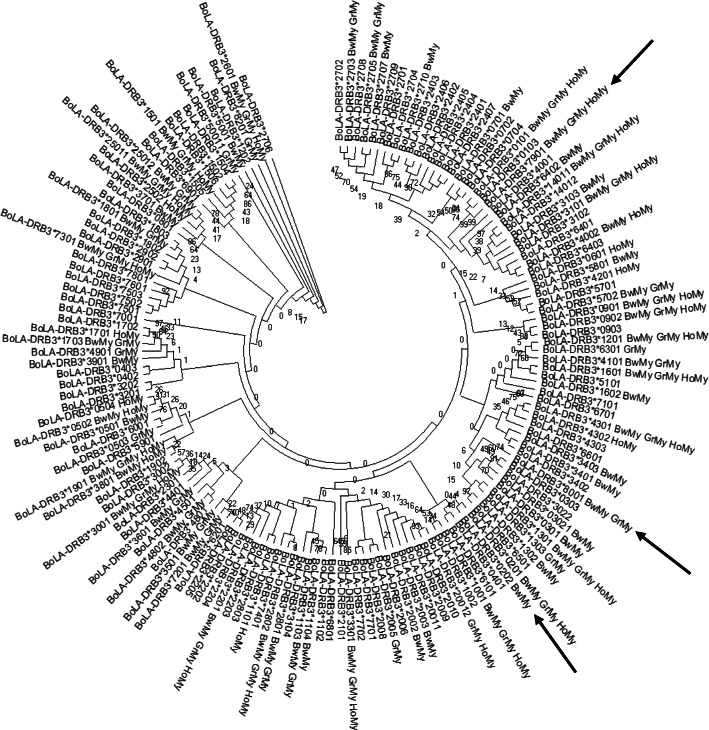
Neighbor-joining (NJ) tree constructed from the 270 bp nucleotide sequence that includes the β1 domain encoded by all reported *BoLA-DRB3* alleles and the three new ones (BoLA-DRB3*002:02, BoLA-DRB3*079:01 and BoLA-DRB3*080:01). Numbers are bootstrap percentages that support each node. Bootstraping was carried up with 1000 replicates to access the reliability of individual branches. New alleles are indicated with arrows. BwMy = Pyer Sein, GrMy = Shwe Ni, HoMy = Myanmar Holstein-Friesian crossbreed

A Venn diagram was constructed using data obtained in this study and from previous reports [18, 23, 29] which include 102 BoLA-DRB3 alleles. Data were grouped in terms of the breed’s geographical origin as follows: Myanmar native breeds (Pyer Sein and Shwe Ni) and Holstein-Friesian crossbreed; Asian native (Philippine native and Japanese Black); Zebu (Bolivian Nellore, Bolivian Gir, Peruvian Nellore-Brahman and Philippine Brahman); and European (Chilean Hereford, Chilean Black Angus, Chilean Red Angus, Japanese Jersey, Japanese Shorthorn and Japanese Holstein) breeds. Six alleles were not present in any of these breed groups. This analysis revealed that out of the 96 alleles identified in the five cattle groups, only nine were detected in the Myanmar native breeds (Fig. 2a), three of which exhibited gene frequencies that were higher than 0.5% (Fig. 2b). Two other variants were only present in Myanmar native breeds and the Holstein-Friesian crossbreed. Together, these 11 alleles represent about 15% of the 73 alleles detected in the Myanmar native cattle and Holstein-Friesian crossbreed. Twenty-six other alleles were only found in Myanmar cattle populations and Asian native or Zebu breeds, or a combination of these groups. In addition, the *BoLA-DRB3* NJ tree including all the previously reported alleles and the three new variants ones showed that the variants detected in Myanmar cattle populations were interspersed among the various clusters (Fig. 3).

Three, six and five alleles appeared with frequencies of > 5% in Pyer Sein, Shwe Ni and Holstein-Friesians, respectively. Three of these high-frequency (> 5%) alleles (BoLA-DRB3*002:01, *017:03 and *022:01) were common in at least two out of three Myanmar populations (Table 1). These common alleles accounted for a low proportion of the cumulative gene frequencies (19.02, 34.78 and 43.13% in Pyer Sein, Shwe Ni and Holstein-Friesians, respectively), revealing an even gene frequency distribution (Fig. S1).

### Nucleotide and amino acid diversity in the *BoLA-DRB3* alleles found in Myanmar cattle populations

The results of genetic diversity at the DNA and amino acid levels obtained for Myanmar cattle breeds and for breeds previously reported are shown in Table 3. The π values within Pyer Sein, Shwe Ni and Holstein-Friesian crossbreed were 0.090, 0.080 and 0.080, respectively, while the mean number of pairwise differences was 20.96, 17.89 and 20.09, respectively. These nucleotide diversity values all fall within the upper end of the range reported (π_range_ = 0.068–0.083; NPD_range_ = 16.31–20.04) for other bovine breeds when using PCR-SBT genotyping methods [18, 22, 23, 26, 29]. The average d_N_ and d_S_ substitutions in Myanmar cattle breeds was calculated across *BoLA-DRB3* exon 2 and the antigen-binding site (ABS). As expected, the d_N_/d_S_ ratio was higher when only the ABS was analyzed. As shown in Table 3, the values obtained in Myanmar cattle were similar to those estimated for other cattle breeds (d_N_/d_S total_ = 0.054–0.067; d_N_/d_S ABS_ = 0.247–0.282).

**Table 3:** Nucleotide diversity (π), mean number of pairwise differences (NPD) and mean number of non-synonymous (d_n_) and synonymous (d_s_) nucleotide substitutions per site. ABS = antigen-binding site

Breed	π	NPD	Total			ABS		
			ds	dn	dn / ds	ds	dn	dn / ds
Pyer Sein^a^	0.090	20.96	0.038	0.096	2.53	0.127	0.387	3.05
Shwe Ni^a^	0.080	17.89	0.039	0.098	2.51	0.122	0.397	3.25
Holstein-Friesian crossbreed^a^	0.080	20.09	0.043	0.099	2.30	0.141	0.4	2.84
Yacumeño ^b^	0.078	19.59	0.036	0.099	2.75	0.128	0.391	3.05
Hartón del Valle ^b^	0.076	19.00	0.029	0.096	3.31	0.109	0.386	3.54
Bolivian Nellore ^c^	0.070	17.54	0.035	0.097	2.77	0.117	0.388	3.32
Bolivian Gir ^c^	0.078	19.45	0.038	0.096	2.53	0.133	0.385	2.89
Nellore x Brahman ^c^	0.068	16.95	0.039	0.097	2.49	0.128	0.376	2.94
Japanese Holstein ^d^	0.079	19.86	0.038	0.096	2.53	0.132	0.393	2.98
Japanese Shorthorn ^d^	0.079	19.80	0.041	0.097	2.37	0.128	0.41	3.20
Japanese Jersey ^d^	0.073	16.31	0.041	0.099	2.41	0.122	0.402	3.30
Japanese Black ^d^	0.071	18.56	0.043	0.096	2.23	0.139	0.365	2.63
Chilean Hereford ^e^	0.070	17.41	0.033	0.098	2.97	0.112	0.46	4.11
Chilean Black Angus ^e^	0.077	19.17	0.037	0.097	2.62	0.123	0.385	3.13
Chilean Red Angus ^e^	0.080	20.03	0.041	0.097	2.37	0.124	0.389	3.14
Philippine Brahman ^f^	0.080	20.04	0.04	0.096	2.40	0.133	0.379	2.85
Philippine Native ^f^	0.083	19.60	0.036	0.096	2.67	0.12	0.381	3.18

^a^ Present work; ^b^ Giovambattista et al., [26]; ^c^Takeshima et al., [29]; ^d^Takeshima et al., [18]; ^e^Takeshima et al., [22, 23]; and ^f^Takeshima et al., [21]

### Gene diversity, HWE, and neutrality testing of *BoLA-DRB3* variants found in Myanmar cattle populations

As mentioned above, n_a_ ranged from 33 in the Holstein-Friesian crossbreed to 58 in the Pyer Sein native breed, while h_e_ and h_o_ were both higher than 0.86 in all three populations (Table 2). These indexes are evidence of high diversity values for Myanmar cattle populations, which is similar to the results reported for other bovine breeds which have been evaluated by PCR-SBT, and characteristic of *MHC class II DR* genes [18, 22, 23, 26, 29]. When we evaluated the populations using the HWE test, two of the three Myanmar populations were in equilibrium, while native breed Shwe Ni significantly deviated from its theoretical values (Table 2), probably because of the significant proportions of homozygous animals found in this study (F_IS_ = 0.1197, *p* <  0.0001). As demonstrated in Table 2, other breeds have also been seen to be in disequilibrium, as a result of an excess or deficit in the proportion of homozygous animals within the population.

It is widely accepted that the genetic diversity of *MHC class II* genes can be maintained by balancing selection. Thus, we performed a Slatkin’s exact neutrality test (Table 2) to evaluate this phenomenon in our populations. The *BoLA-DRB3* gene frequency profile in both the Pyer Sein and Shwe Ni cattle showed an even distribution (*p* = 0.006 and 0.010, respectively), consistent with the theoretical proportion expected under balancing selection pressures as opposed to positive or neutral selection (*p* > 0.025). A similarly even *BoLA-DRB3* gene frequency was observed in other cattle breeds, including Japanese Black, Yacumeño Creole and Bolivian Gir. Conversely, *BoLA-DRB3* gene frequency distributions in the Holstein-Friesian crossbreed were more compatible with neutral selection, which is similar to the results obtained for the majority of the cattle breeds analyzed to date (Table 2).

### *BoLA-DRB3* genetic structure and levels of population differentiation in Myanmar cattle

**Fig. 4:**
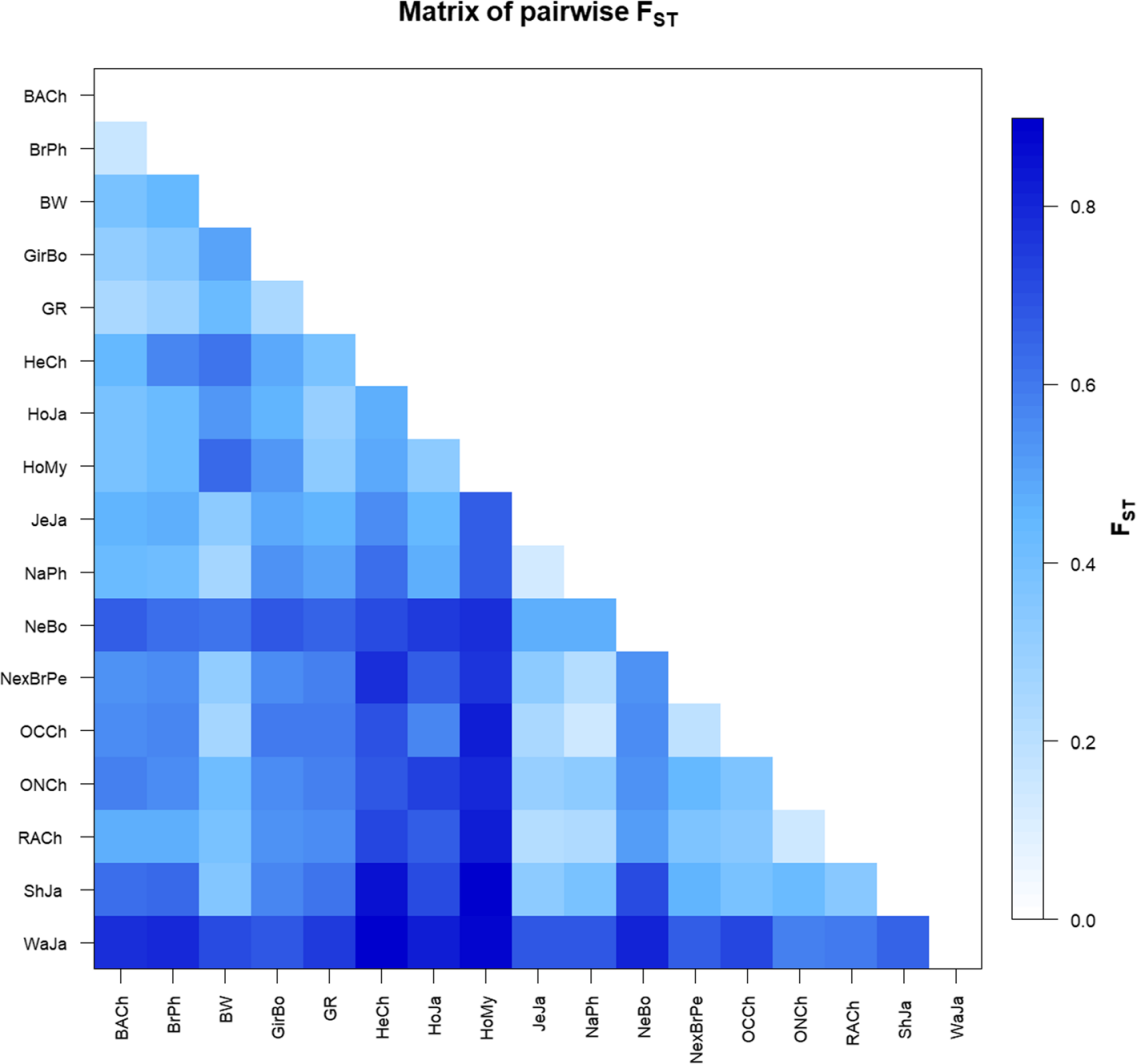
Graphic representation of calculated F_ST_ between population pairs using an R function pairFstMatrix.r. BW = Pyer Sein, GR = Shwe Ni, HoMy = Myanmar Holstein-Friesian crossbreed, NeBo = Bolivian Nellore, GirBo = Bolivian Gir, BrxNe = Peruvian Brahman × Nellore crossbreed, HoJa = Japanese Holstein, ShJa = Japanese Shorthorn, JeJa = Japanese Jersey, WaJa = Japanese Black, HeCh = Chilean Hereford, BACh = Chilean Black Angus, RACh = Chilean Red Angus, ONCh = Chilean Overo Negro, OCCh = Chilean Overo Colorado, NaPh = Philippine native, and Philippine Brahman (BrPh)

The average *F*_*ST*_ analysis showed a low level of genetic differentiation between Myanmar native breeds (F_ST_ = 0.003), similar to those estimated in Holstein populations (0–0.0067) [23]. F_ST_ values between the native breeds and the Holstein-Friesian crossbreed varied from 0.019 to 0.021. These values were within the range estimated for differences within Taurine or Zebu breeds and lower than those obtained when comparing breeds from different groups (Fig. 4 and Table S2).

The average *F*_ST_ values across all Myanmar Holstein-Friesian and native breed populations were 0.0136 and 0.0121, respectively (*p* <  0.001). Significant differences were observed in nine out of the fifteen native and one out of six Holstein-Friesian crossbreed populations (*p* <  0.05). In addition, *F*_ST_ values for comparisons between Myanmar native breeds ranged between 0.003 and 0.024 and between 0 and 0.031 for Myanmar Holstein-Friesian crossbreed populations (Table S3a and b). As mentioned above, similar genetic distance values were observed among Holstein populations from different countries [23].

### Genetic differentiation of *BoLA-DRB3* alleles in Myanmar breeds. Comparison with zebu and taurine breeds

**Fig. 5:**
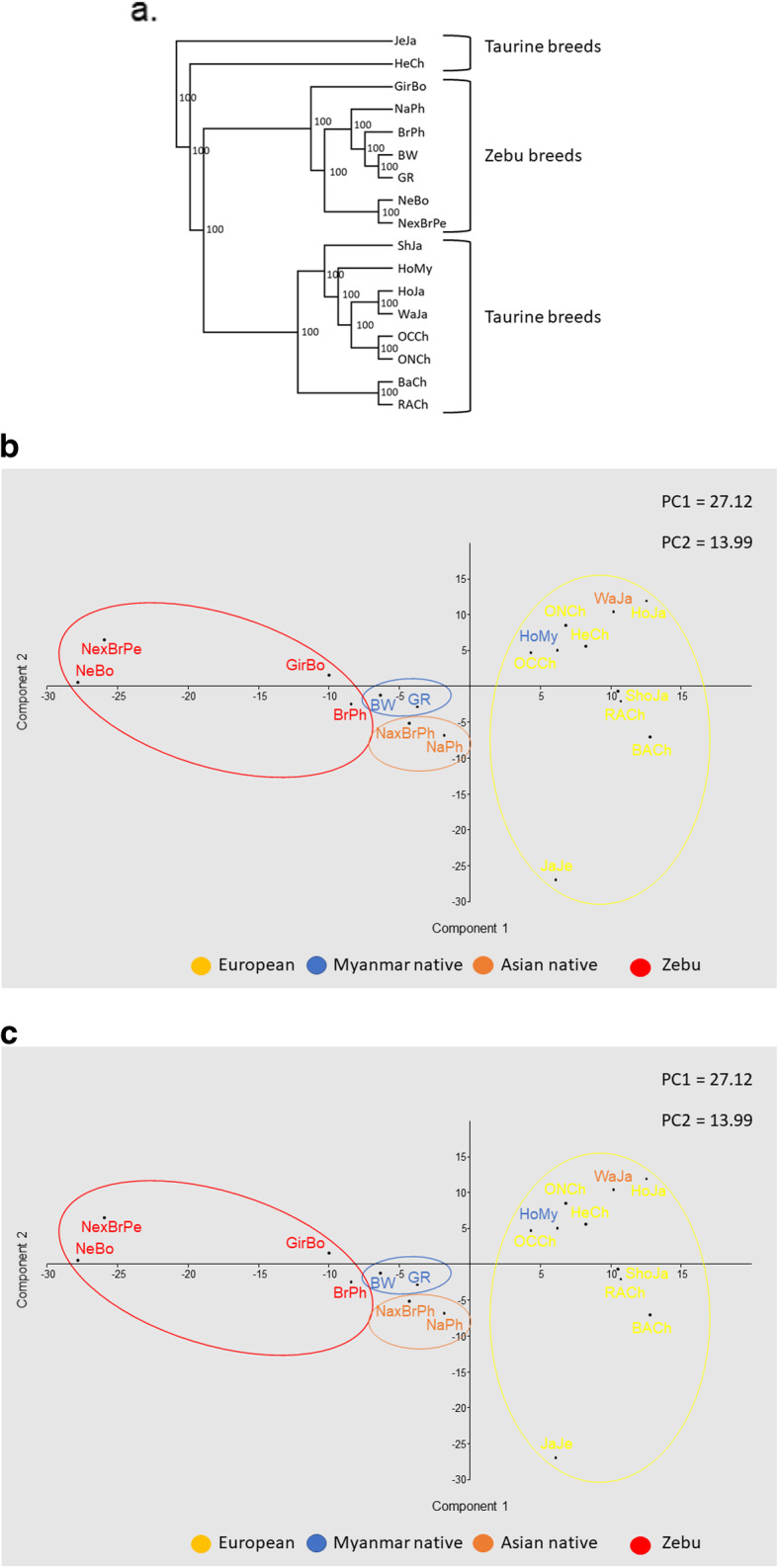
**a** UPGMA dendrogram constructed from a matrix of D_A_ genetic distances. **b** and **c** principal component analysis of allele frequencies from the *BoLA-DRB3* gene in 18 populations. BW = Pyer Sein, GR = Shwe Ni, HoMy = Myanmar Holstein-Friesian crossbreed, NeBo = Bolivian Nellore, GirBo = Bolivian Gir, and BrxNe = Peruvian Brahman × Nellore crossbreed, HoJa = Holstein, ShJa = Japanese Shorthorn, JeJa = Jersey, WaJa = Japanese Black, HeCh = Chilean Hereford, BACh = Chilean Black Angus, RACh = Chilean Red Angus, ONCh = Chilean Overo Negro, OCCh = Chilean Overo Colorado, NaPh = Philippine Native, BrPh = Philippine Brahman and NaxBrPh = Native x Brahman Philippine crossbreed

First, *BoLA-DRB3* allele frequencies from Myanmar and previously reported breeds included in our dataset were used to generate Nei’s D_A_ and D_S_ genetic distance matrices. Then, dendrograms were constructed from these distance matrices using UPGMA and NJ algorithms. All trees revealed congruent topologies, which were consistent with the historical and geographical origin of the breeds. As expected, this tree revealed two main clusters which included most of the Taurine and Zebuine breeds (Fig. 5a), with Japanese Jersey and Chilean Hereford located outside of these clusters. The Holstein-Friesian crossbreed fell into the Taurine cluster, while the Myanmar native breeds were located in a sub-cluster within the Zebuine cluster close to Philippine populations.

Second, we used *BoLA-DRB3* allele frequencies to perform three PCA analyses among breeds. In these PCA, the first two components accounted for 41.11% of the data variability. The first PC accounted for 27.12% of the total variance and, as shown in a previous study [29], first component clearly exhibited a differentiation pattern between Zebu (negative values) and Taurine (positive values) breeds, while native breeds from Myanmar and the Philippines were located near the origin of the plot (Fig. 5b). The first PC was primarily determined by differences in the frequency of the same alleles reported by Takeshima et al. [29]. The second PC explained 13.99% of the total variation and showed a gradient among Taurine breeds, with Japanese Black and Japanese Jersey located at opposite ends. Furthermore, this component discriminated between Myanmar and Philippine native breeds. The second PC was identical to PC1 reported in the study mentioned above [29]. Finally, the third PC accounted for 13.64% of the variance and allowed the differentiation of Chilean Hereford cattle from other Taurine breeds. As shown in Fig. 5b and c, the Myanmar Holstein-Friesian crossbreed was located within the Taurine cloud but in an intermediate position between the Japanese Holstein and Myanmar native breeds, supporting the presence of the same level of gene introgression in Myanmar Holstein populations, which is also supported by the presence of unique *BoLA-DRB3* alleles within these populations. These PCA results agree with the overall clustering observed after NJ or UPGMA tree construction.

In addition, we analyzed protein pockets (pocket 1, pocket 4, pocket 6, pocket 7, and pocket 9) involved in the antigen-binding function of the *MHC* complex using PCA. As shown in Fig. S2 a-e, only the distribution pattern of pocket 4 was similar to the PCAs created using the allelic frequency data, while PCA of the remaining pockets did not exhibit a spatial distribution related to the geographical or historical origin of the breeds. The position of the Myanmar native breeds in pocket 4 was the result of positive PC1 and PC2 values for the presence of amino acid motifs GFDQKEV, SYDRENY, SFDREYY, SFDDEAY, KFDRAAY, and GYDREYY (amino acid positions 13, 26, 28, 70, 71, 74 and 78). Also, pocket 1 showed quite similar distribution pattern, but for PC2.

**Fig. 6:**
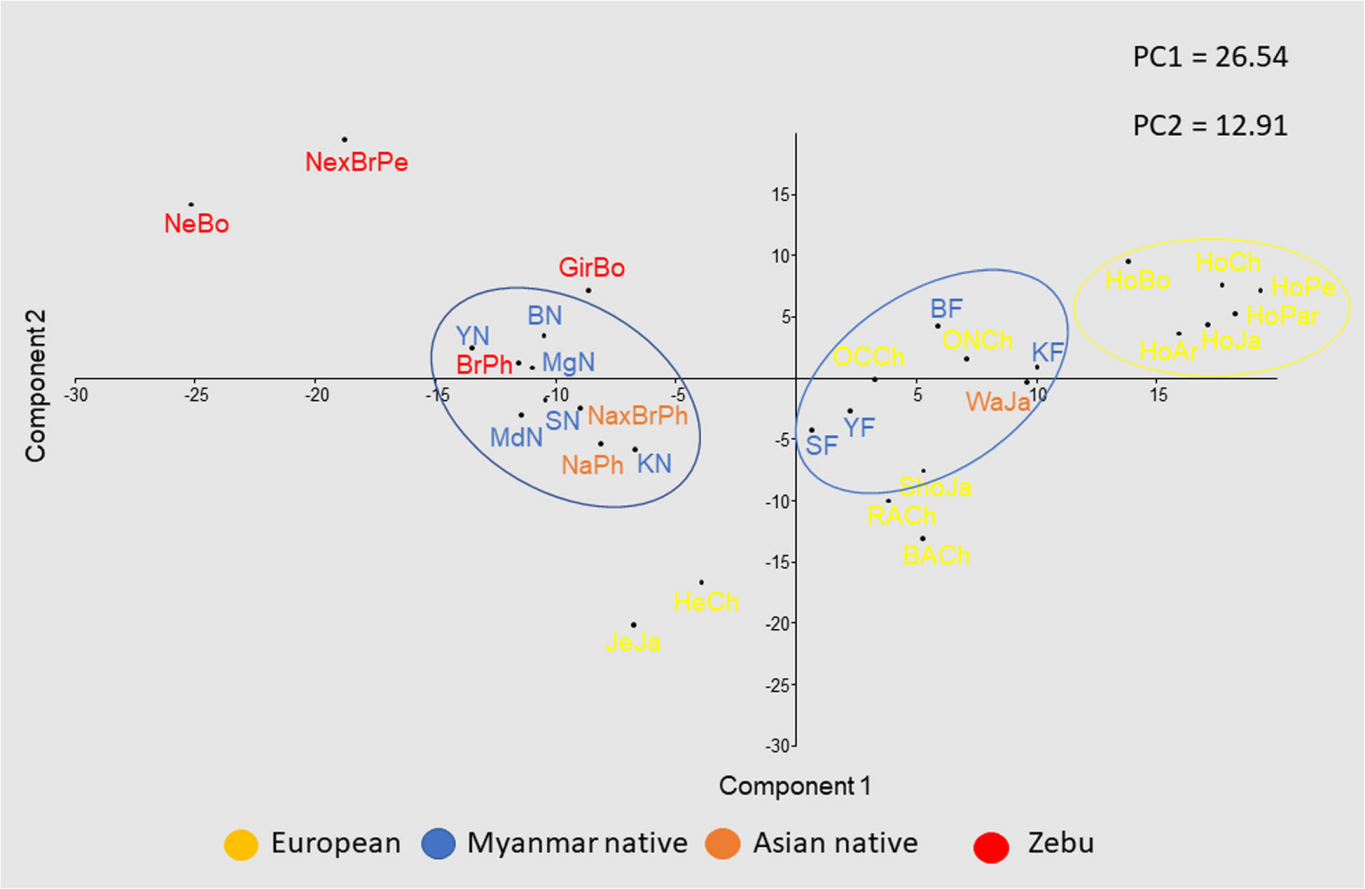
Principal components analysis of allele frequencies from the *BoLA-DRB3* gene in six Myanmar native (KN = Kayin, BN = Bago, SN = Sagaing, MdN = Mandalay, MgN = Magway, and YN = Yangon), four Myanmar Holstein-Friesian crossbreed (KF = Kayin, BF = Bago, SF = Sagaing, and YF = Yangon) from analyzed Myanmar State and regions, and 15 breeds/crossbreeds. NeBo = Bolivian Nellore, GirBo = Bolivian Gir, and BrxNe = Peruvian Brahman × Nellore crossbreed, HoJa = Holstein, ShJa = Japanese Shorthorn, JeJa = Jersey, WaJa = Japanese Black, HeCh = Chilean Hereford, BACh = Chilean Black Angus, RACh = Chilean Red Angus, ONCh = Chilean Overo Negro, OCCh = Chilean Overo Colorado, NaPh = Philippine Native, BrPh = Philippine Brahman, and NaxBrPh = Native x Brahman Philippine crossbreed

Finally, PCA was performed at the Myanmar population level, to evaluate the impact of the ten sampling sites (six for native breeds and four for Holstein-Friesians) on our results. This analysis showed that Myanmar native populations grouped in a narrow cluster that diverged clearly from the Myanmar Holstein-Friesian crossbred populations (Fig. 6), in agreement with the F_ST_ analysis described above. Furthermore, PCA showed that some Myanmar native populations (Bago, Mandalay and Yangon) seemed to be closer to Zebu breeds (Nellore, Gir and Brahman), while others (Kayin, Magway and Sagaing) were more closely related to the Philippine native breeds. However, PCA results at the Myanmar population level did not show a clear correlation between the genetic relationship of *BoLA-DRB3* alleles and geographical distribution. By contrast, Myanmar Holstein-Friesian populations showed a more dispersed distribution when compared to the compact cloud reported for Holstein populations from other countries [23], which may be the result of the differences in the degree of admixture between these populations.

## Discussion

In this study, we carried out the first genetic characterization of the *BoLA-DRB3* gene in two Myanmar native breeds and the Myanmar Holstein-Friesian crossbreed population using PCR-SBT. This analysis allowed us to detect 71 alleles, including three new variants. Analyses of D-loop mitochondrial haplotypes revealed a large percentage of novel haplotypes in Myanmar native breeds, suggesting that these indigenous populations may possess novel polymorphisms throughout the genome of these native breeds [32, 33]. The *BoLA-DRB3* allele NJ tree, generated using the nucleotide sequence of the β1 domain of all the reported alleles and the three new ones, evidenced that the variants detected in Myanmar cattle populations were not grouped in specific clusters of the dendrogram, but were interspersed along the tree. Similar results were reported in South American native cattle breeds [26] and agree with the ancient origin of the MHC alleles proposed by the trans-specific theory of MHC alleles [34]. According to this theory, these alleles originated before cattle domestication and speciation, meaning that many variants will be common across species and geographies. This is supported by the fact that BoLA-DRB3*1303 (gi|1,261,491,414|gb|KY094633.1|) which was detected in this work was previously described by a study focused on Indonesian *Bos javanicus*.

In order to understand the distribution of the *BoLA-DRB3* alleles across breeds, a Venn diagram was constructed. This analysis demonstrated that 11 *BoLA-DRB3* alleles were only detected in Myanmar cattle populations, at least for the breeds included in this analysis. Three of these alleles were entirely unique variants, described in this study (BoLA-DRB3*002:02, BoLA-DRB3*079:01 and BoLA-DRB3*080:01). It is worth noting that a review of the IPD–MHC database showed that some variants had been previously detected in other Asian breeds (BoLA-DRB3*031:03, *058:01 and *064:02 in Chinese yellow cattle, BoLA-DRB3*013:03 in Korean Hanwoo cattle and BoLA-DRB3*049:01 in the Indian Red Sindhi); African Zebu breeds (BoLA-DRB3*038:01 in Ethiopian Arsi and BoLA-DRB3*013:03 in Sudanese Baggara) and other Asian Bos species, including BoLA-DRB3*013:03 in Indonesian *Bos javanicus*. In previous studies privative alleles were also identified in native breeds from Asia and South America [21, 26, 28]. In addition, some alleles detected in Creole cattle have also been detected in African breeds (Boran, N’Dama, Ethiopian Arsi, and Gudali) and *Bos indicus* (Brahman). Together, these results show that *BoLA-DRB3* variants could be classified using the following categories: worldwide geographical distribution, present only in one major bovine type (Taurus or Indicus), located in a geographical region, and detected in only one or a few related breeds. The current geographical allele distribution could be the result of several factors, including breed origin, founder effect, natural or artificial selection and recent or historical gene introgression.

The large number of *BoLA-DRB3* variants indicate a high level of genetic diversity in Myanmar cattle populations. High genetic variability was not unexpected for the *BoLA-DRB3* locus, with the values recorded in this study falling in line with observations made in Philippine native and Hanwoo cattle breeds [21, 28]. A high level of diversity was also observed in DNA sequences when nucleotide diversity and mean number of pairwise differences were estimated. In addition, a higher number of d_n_ than d_s_ changes (especially in antigen peptide binding sites) were found in this population which is also similar to the findings reported for other cattle breeds. Different mechanisms for the maintenance of the high diversity of MHC loci have been proposed, including balancing selection (i.e., overdominance and negative frequency dependence) [35–37].

The neutrality test showed that both Myanmar indigenous breeds had an even allele frequency distribution, while the most common allele (> 0.5) accounted for only 19.02% of alleles in Pyer Sein and 34.78% of alleles in Shwe Ni. These results are compatible with the hypothesis of balancing selection rather than positive selection against one or more variants or neutral selection strategies. This finding has also been reported in Yacumeño creole cattle as well as in other cattle breeds (Bolivian Gir, Japanese Jersey and Japanese Black) [26].

Takeshima et al. [38] reported a significant deviation from HWE in class II *BoLA-DQA1* genes in Japanese Holstein cows with mastitis caused by *Escherichia* or *Streptococcus* bacteria. Similar results were observed in human, model and non-model species [37]. This evidence was interpreted using the overdominance selection theory, which proposes that heterozygous individuals recognize a broader spectrum of foreign antigens than homozygous ones, thereby increasing their fitness and selective advantage against pathogens [39, 40]. In this study, only the Shwe Ni native breed showed a significant deviation from HWE theorical proportions resulting from an excessive proportion of homozygotic animals within the population. However, none of the Myanmar cattle populations evidenced a significant excess of heterozygotes. These results agree with data observed in other cattle populations exhibiting HWE or excessive numbers of homozygotes, and could be the consequence of low values for overdominance selection coefficients in respect of the stochastic forces (e.g., genetic drift, inbreeding), population structure (Whalund effect) and the low-resolution power of HWE methods [40].

The genetic structure and breed relationships of *BoLA-DRB3* polymorphisms were studied using an F_ST_ index, PCA and dendrograms. Together, these analyses showed that Myanmar native breeds, which are mainly characterized by their geographical location and coat color, exhibited a low level of genetic divergence and tended to be closely related in both PCA and dendrograms. These results were reinforced when PCA was performed at the population level, and was able to demonstrate that there was a low level of genetic differentiation among Myanmar cattle populations (F_ST_ = 0.0121). These breeds grouped in a narrow cluster with a low level of genetic distance between them. Furthermore, no correlation between genetic and geographical distance for the *BoLA-DRB3* alleles was observed among these populations. In previous studies, assignation to the Chinese yellow cattle populations using microsatellites and SNP markers did not allow researchers to distinguish unique genetic differences for each of the Chinese breeds, and individuals could not be correctly assigned to their breed based on their genotype, despite two specific yellow cattle genetic profiles being described [41, 42]. For these reasons native cattle breeds from Eastern Asia are usually described based on their geographical region, without breeding associations or herd books that create breeding barriers, mainly characteristic of the European breeds. These types of analysis that allow to identify genetic entities are important in the development and design of conservation programs created to protect these native cattle breeds.

In addition, PCA and dendrograms showed that Myanmar native breeds were located close to Philippine populations and fell into a sub-cluster within the Zebuine cluster. Furthermore, PCA showed that some Myanmar native populations (Bago, Mandalay, and Yangon) seemed to be closer to some Zebu breeds (Gir, and Brahman), while others (Kayin, Magway, and Sagaing) were more closely related to the Philippine native breeds. This is in agreement with Myanmar’s location in South East Asia, where it shares borders with Bangladesh and India to the Northwest, China to the Northeast, Laos to the East and Thailand to the Southeast, and the fact that Myanmar native breeds are phenotypically humped cattle. Studies based on blood proteins and SRY gene polymorphisms support our conclusion that these indigenous cattle are zebu type animals [43, 44]. In addition, Chen et al. [32] and Lwin et al. [33] showed that Myanmar native cattle had zebu mitochondrial haplotypes, confirming our hypothesis.

When breed relationships were studied using the gene frequencies described for the amino acid motifs found in the ABS pockets implicated in antigen-binding function, only the PCA from pocket 4 demonstrated a similar distribution pattern to the PCA pattern developed using allelic frequency data. By contrast, the remaining PCAs did not exhibit a spatial distribution compatible with the geographical or historical origin of the analyzed breeds. The position of the Myanmar native breeds in the pocket 4 PCA was as a result of their positive PC1 and PC2 values for amino acid motifs GFDQKEV, SYDRENY, SFDREYY, SFDDEAY, KFDRAAY, and GYDREYY. Previous studies showed that pocket 4 has been found to be important for the binding of peptides may be due to this pocket being located in the center of the PBC [45]. In addition, it has been reported in cattle that immune responses and disease resistance is significantly related to differences in the pocket 4 motif [45, 46]. Takeshima et al. [29] reported that PCAs based on allelic frequencies in the amino acid motif of the ABS pockets showed a clear differentiation between Taurine and Zebu breeds, because of an enrichment for particular amino acid motifs in specific pockets. Further studies involving structural modeling and molecular simulation of the BoLA-DRβ protein are needed to elucidate whether these differences play a role in its function.

The Myanmar Holstein-Friesian crossbreed was located within the Taurine cloud but in an intermediate position between Japanese Holstein and Myanmar native breeds, probably as a consequence of gene introgression in Myanmar Holstein-Friesian cattle, which is also supported by the presence of *BoLA-DRB3* alleles unique to the native and Holstein-Friesian breeds from Myanmar. The average level of genetic differentiation observed among the Myanmar Holstein-Friesian crossbreed populations (F_ST_ = 0.0136) was higher than those previously reported in Holstein populations from other countries (F_ST_ = 0.009) [23]. Its distribution was more dispersed in the PCA when compared with the more compact cloud reported for other Holstein populations [23]. These differences are likely the result of the different degree of admixture between this crossbreed and the Myanmar native cattle.

Holstein cattle are mainly raised in temperate and cold regions because they are sensitive to heat stress and susceptible to tropical disease. However, this breed has been introduced to tropical and subtropical countries several times with varying degrees of success, and it is possible to find Holstein crossbreed populations in these environments. In 1978, pregnant Holstein cows from New Zealand and Australia were imported to Myanmar to improve the local dairy cows [47]. Later, semen was introduced from North America and Europe [47, 48]. The present *BoLA-DRB3* gene results would indicate that foreign Holstein cattle could be crossed with local cattle to create an adapted Holstein crossbreed population. To support this, four *BoLA-DRB3* alleles (BoLA-DRB3*022:01, BoLA-DRB3*026:01, BoLA-DRB3*031:01 and BoLA-DRB3*079:01), including the novel BoLA-DRB3*079:01 variant, were present in the Myanmar Holstein-Friesian population. Notably, these alleles were common in the Myanmar native cattle population, but they had a very low frequency or were absent in Holstein populations from temperate or cold regions. This is consistent with the results reported by Takeshima et al. [23], which demonstrated that Bolivian Holsteins, raised in the humid subtropical plains of Santa Cruz (Bolivia), diverged from cattle bred in temperate and cold geographical regions. In addition, the Bolivian Holstein population was found between Yacumeño Creole cattle and the compact cloud of Holstein populations when evaluated as part of a larger data set and shared common alleles with this Creole breed. In this sense, the introgression of genes from locally adapted native cattle in Holstein-like populations following natural selection may have contributed to increased fitness in tropical and subtropical regions.

## Conclusions

This study is the first to report the genetic diversity of the *BoLA-DRB3* gene in two native breeds and one exotic cattle crossbreed from Myanmar, using the PCR-SBT assay. These results revealed the presence of three new alleles and demonstrated a high degree of genetic diversity in this gene, which could contribute to the adaptation of these breeds to the harsh subtropical environmental of Myanmar. Furthermore, dendrograms and PCA showed that Myanmar native breeds were closely related to one another and to the Philippine native breeds at this locus. Therefore, this study increases our knowledge of the worldwide variability of cattle *BoLA-DRB3* genes, an important locus for immune response and protection against pathogens.

## Methods

### Sample populations and genomic DNA extraction

Blood samples were obtained from 294 cattle belonging to the Myanmar Pyer Sein (*N* = 163), Shwe Ni (*N* = 69) native breeds and the Holstein-Friesian crossbreed (*N* = 62), distributed across six regions of Myanmar (Bago, *n* = 38; Sagaing, *n* = 77; Mandalay, *n* = 46; Magway, *n* = 46; Kayin, *n* = 43; Yangon, *n* = 44) (Table 1; Fig. S3). Blood samples were randomly collected from adult animals according to the availability and permission of local farmers in the respective regions. A database that included 2428 *BoLA-DRB3* genotypes from European, Zebuine and Native breeds was also included (Table S1) [18, 23, 26, 29]. These genotypes data were used for recalculating the genetic diversity indexes at alleles and sequence level in breeds details in Table S1 for further comparison with Myanmar results. The genomic DNA was extracted from whole blood using a PureLink Genomic DNA Mini Kit (Invitrogen, Carlsbad, CA, USA), according to the manufacturer’s instructions.

### *BoLA-DRB3* genotyping

*BoLA-DRB3* alleles were genotyped using PCR-SBT. Briefly, *BoLA-DRB3* exon 2 was amplified using PCR according to the method described by Takeshima et al. [20]. The PCR fragments were purified using an ExoSAP-IT PCR Product Purification Kit (USB Corp., Cleveland, OH) and sequenced using the ABI PRISM BigDye Terminator Cycle Sequencing Ready Reaction Kit (Applied Biosystems, Foster City, CA). The raw sequence data were analyzed using Assign 400ATF ver. 1.0.2.41 software (Conexio Genomics, Fremantle, Australia).

### Statistical analyses

#### Measures of genetic variability

Allele frequencies and the observed number of alleles (n_a_) were obtained by direct counting. The distribution of alleles across breeds was represented by a Venn plot created using the R package ‘VennDiagram’ [49]. The observed (h_o_) and unbiased expected (h_e_) heterozygosity of the *BoLA-DRB3* locus were estimated according to the values and assumptions described in Nei [50] using the Arlequin 3.5 software for population genetic analyses [51]. Potential deviations from Hardy-Weinberg equilibrium (HWE) were estimated using F_IS_ statistics [52] for each breed calculated using the exact test included in Genepop 4.7 software [53]. The Ewens–Watterson–Slatkin exact test of neutrality was estimated using the method described by Slatkin [54] and implemented in the Arlequin 3.5 program.

#### Genetic structure and population differentiation

Genetic structure and genetic differentiation among the breeds were assessed using Wright’s F_ST_ statistics, calculated using the variance-based method of Weir and Cockerham [52]. This parameter was estimated using Arlequin 3.5 and Genepop 4.7 software. The F_ST_ values were represented graphically using the pairFstMatrix.r function implemented in the Arlequin 3.5 software.

#### Analysis of the genetic relationship between breeds

To condense the genetic variation at the *BoLA-DRB3* locus, allele frequencies were used to perform a Principal Component Analysis (PCA) according to the Cavalli-Sforza [55] method, implemented in Past software [56]. Nei’s standard genetic distances Ds [57] and D_A_ [58] were calculated from allele frequencies to perform a cluster analysis using the unweighted pair-group method with arithmetic mean (UPGMA) [59] and the neighbor-joining algorithm (NJ) [60]. Confidence intervals for the groupings were estimated by bootstrap re-sampling of the data using 1000 replicates. Genetic distances and trees were computed using the Populations 1.2.28 software [61]. The trees were then visualized using TreeView [62].

#### Genetic diversity at the sequence level

Nucleotide diversity (π) and pairwise comparisons of nucleotide substitutions between alleles (defined as the average number of differences between pairs of DNA sequences) were calculated using Arlequin 3.5. The mean number of nonsynonymous (d_N_) and synonymous (d_S_) nucleotide substitutions per site calculated as an average over all sequence pairs was estimated within each group using the Nei-Gojobori model [63] and Jukes–Cantor’s formula. These parameters were estimated using Arlequin 3.5 and MEGA X [64]. The *BoLA-DRB3* allele tree was constructed using a distance matrix based on the NJ method. To test the significance of each of the branches, 1000 bootstrap replicate calculations were performed. The allele tree was constructed using MEGA X software.

## Supplementary information

**Additional file 1: Figure S1.** Cumulative gene frequency plot of *BoLA-DRB3* alleles in Pyer Sein (red), Shwe Ni (violet) and Holstein-Friesian crossbreed (orange) populations.

**Additional file 2: Figure S2.** Principal component analysis of *BoLa-DRB3* gene pocket amino acid motifs frequencies in 18 populations: a. Pocket 1, b. Pocket 4, c. Pocket 6, d. Pocket 7, and e. Pocket 9. BW = Pyer Sein, GR = Shwe Ni, HoMy = Myanmar Holstein-Friesian crossbreed, WaJa = Japanese Black, HoJa = Holstein, ShJa = Japanese Shorthorn, JeJa = Jersey, HeCh = Chilean Hereford, BACh = Chilean Black Angus, RACh = Chilean Red Angus, ONCh = Chilean Overo Negro, OCCh = Chilean Overo Colorado, NaPh = Philippine Native, NaxBrPh = Native x Brahman Philippine crossbreed, BrPh = Philippine Brahman, NeBo = Bolivian Nellore, GirBo = Bolivian Gir, and BrxNe = Peruvian Brahman × Nellore crossbreed.

**Additional file 3: Figure S3.** Geographical distribution of sampling sites of Myanmar cattle that are indicated by a dot.

**Additional file 4: Table S1.** Detailed information about the populations analyzed.

**Additional file 5: Table S2.** Genetic distance between pairs of populations estimated by F_ST_ in (a) six Myanmar native (KN = Kayin, BN = Bago, SN = Sagaing, MdN = Mandalay, MgN = Magway, and YN = Yangon) and (b) four Myanmar Holstein-Friesian crossbreed (KF = Kayin, BF = Bago, SF = Sagaing, and YF = Yangon) populations.

**Additional file 6: Table S3.** Genetic distance between pairs of populations estimated by F_ST_ in (a) six Myanmar native (KN = Kayin, BN = Bago, SN = Sagaing, MdN = Mandalay, MgN = Magway, and YN = Yangon), and (b) four Myanmar Holstein-Friesian crossbreed (KF = Kayin, BF = Bago, SF = Sagaing, and YF = Yangon) populations.

## Data Availability

The data sets supporting the results of this article are included in this manuscript and its additional information files. In addition, the new BoLA-DRB3 variants generated during the current study are available in the IPD-MHC database (accession numbers BoLA09869, BoLA09870, BoLA09871; https://www.ebi.ac.uk/ipd/mhc/) and in the European Nucleotide Archive (ENA) repository (accession numbers LC466585, LC466586, LC466588; https://www.ebi.ac.uk/ena/).

## Abbreviations

BoLA: Bovine leukocyte antigen
MHC: Major histocompatibility complex
ARS: Antigen recognition site
NGS: Next generation sequencing
PCR: Polymerase chain reaction
PCR- SBT: Polymerase chain reaction-sequence-based typing
AlCr: Highland Bolivian Creole cattle
HWE: Hardy-Weinberg equilibrium
ABS: Antigen-binding site
NPD: Mean number pairwise difference
d_n_: Nonsynonymous substitution
d_s_: Synonymous substitutions
n_a_: Number of alleles
h_o_: Observed heterozygosity
h_e_: Expected heterozygosity
PCA: Principal component analysis
PC: Principal component
F_ST_: Fixation index
