# Supplementary material for: Characterization of bovine MHC DRB3 diversity in global cattle breeds, with a focus on cattle in Myanmar
Source: BMC Genet. 2020 Sep 1;21:95. doi: 10.1186/s12863-020-00905-8 (PMC7460757; doi:10.1186/s12863-020-00905-8)
Supplement: Supplementary file 1 — Additional file 1: Figure S1. Cumulative gene frequency plot of BoLA-DRB3 alleles in Pyer Sein (red), Shwe Ni (violet) and Holstein-Friesian crossbreed (orange) populations. [file 12863_2020_905_MOESM1_ESM.docx]

**Fig. S1** Cumulative gene frequency plot of BoLA-DRB3 alleles in Pyer Sein (red), Shwe Ni (violet) and Holstein-Friesian crossbreed (orange).
