# Supplementary material for: Characterization of bovine MHC DRB3 diversity in global cattle breeds, with a focus on cattle in Myanmar
Source: BMC Genet. 2020 Sep 1;21:95. doi: 10.1186/s12863-020-00905-8 (PMC7460757; doi:10.1186/s12863-020-00905-8)

**Fig. S2** Principal component analysis of BoLa-DRB3 gene pocket amino acid motifs frequencies in 18 populations. BW = Pyer Sein, GR = Shwe Ni, HoMy = Myanmar Holstein-Friesian crossbreed, NeBo = Bolivian Nellore, GirBo = Bolivian Gir, and BrxNe = Peruvian Brahman × Nellore crossbreed, HoJa = Holstein, ShJa = Japanese Shorthorn, JeJa = Jersey, WaJa = Japanese Black, HeCh = Chilean Hereford, BACh = Chilean Black Angus, RACh = Chilean Red Angus, ONCh = Chilean Overo Negro, OCCh = Chilean Overo Colorado, NaPh = Philippine Native, BrPh = Philippine Brahman and NaxBrPh = Native x Brahman Philippine crossbreed.

a.


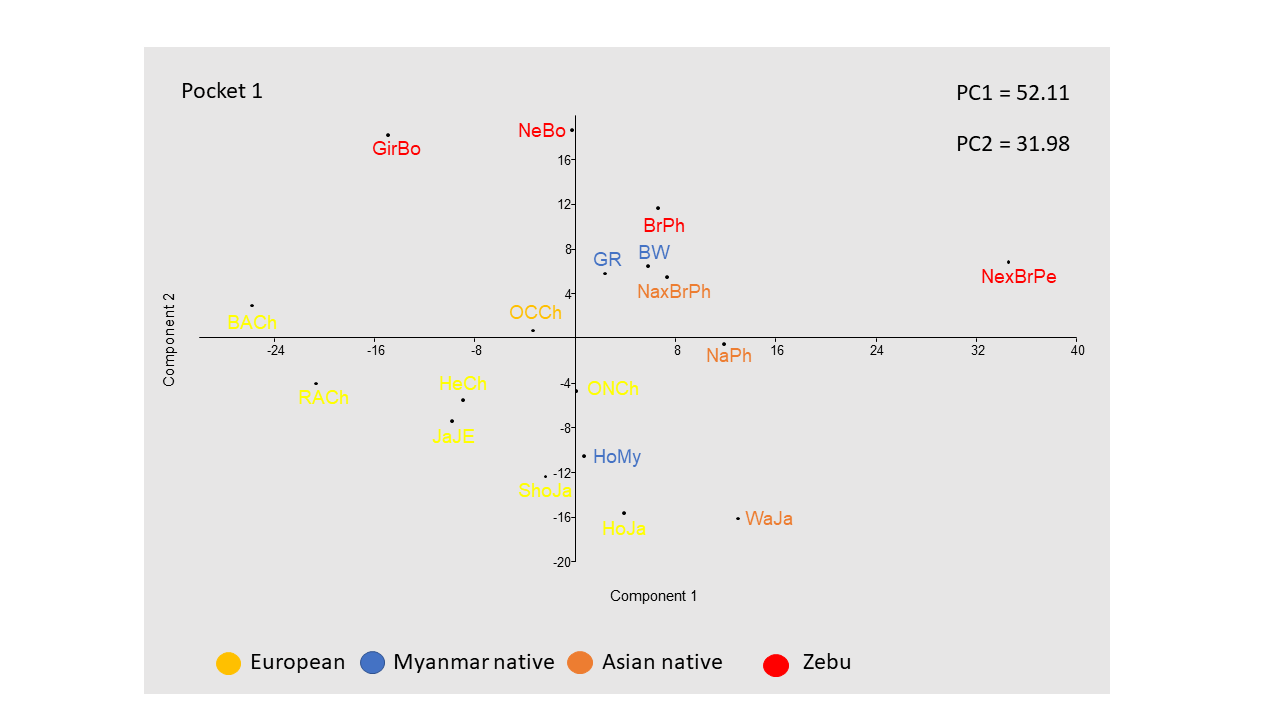


b.


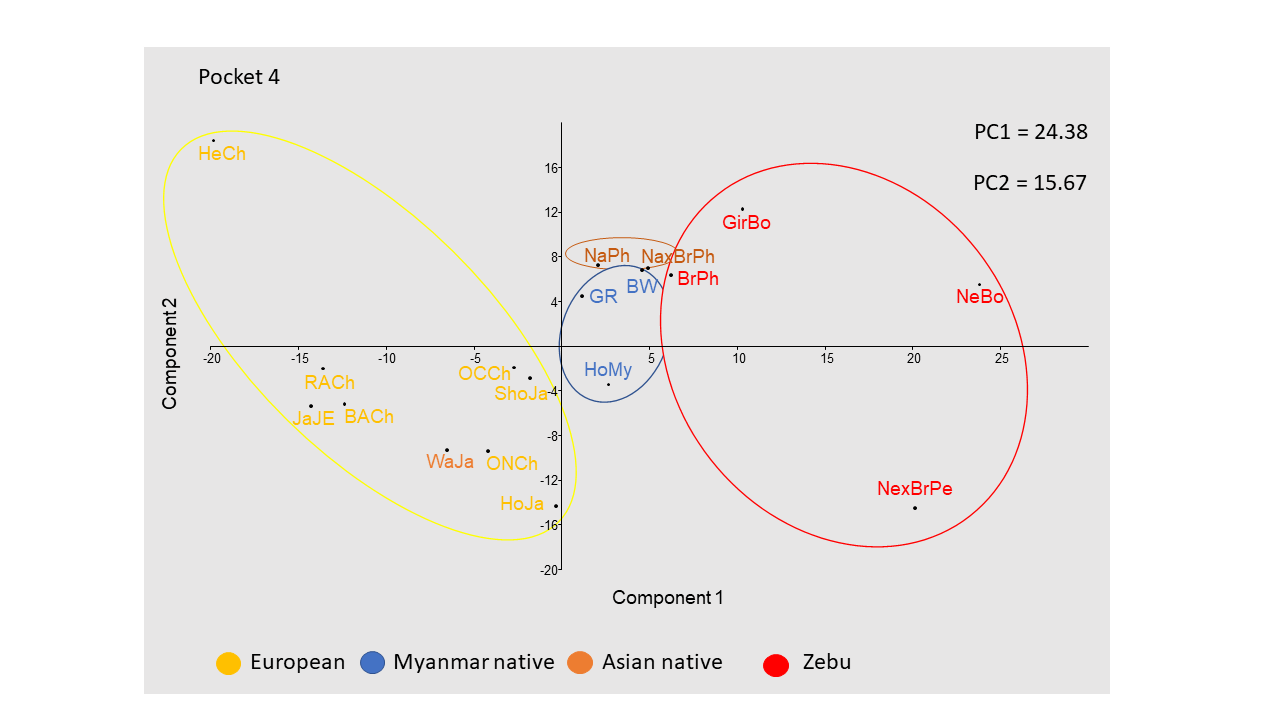


c.


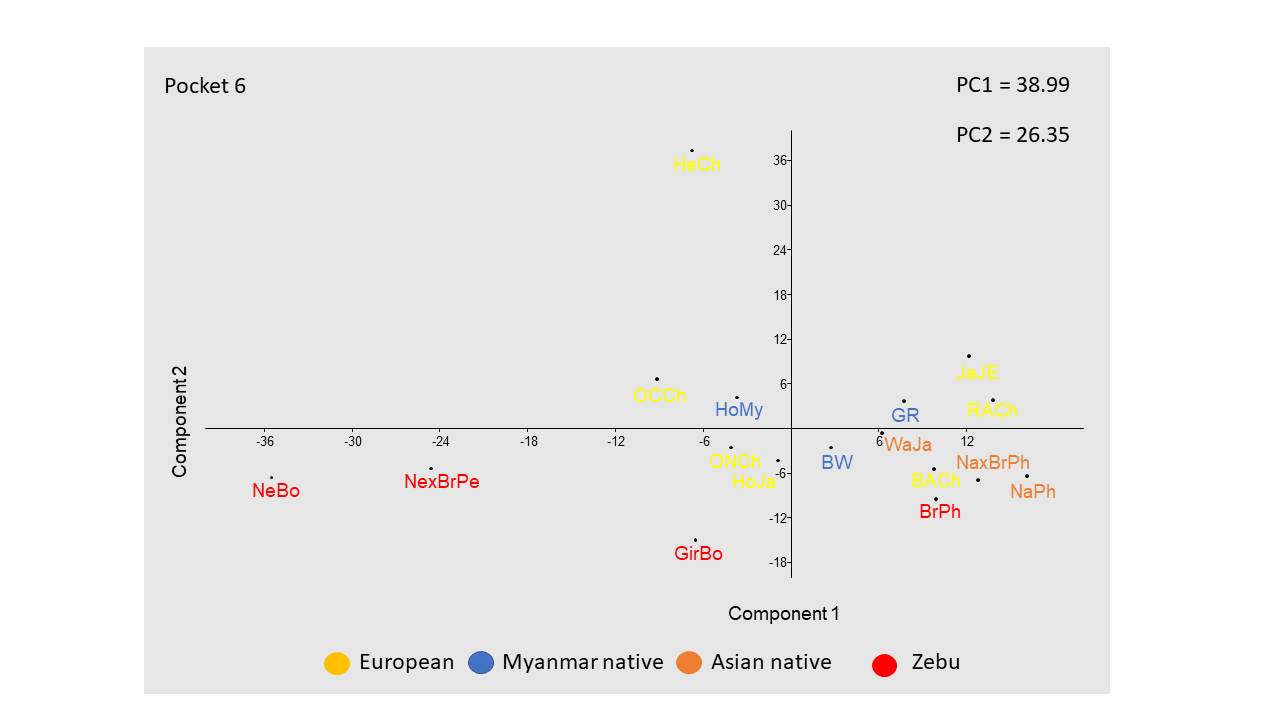


d.


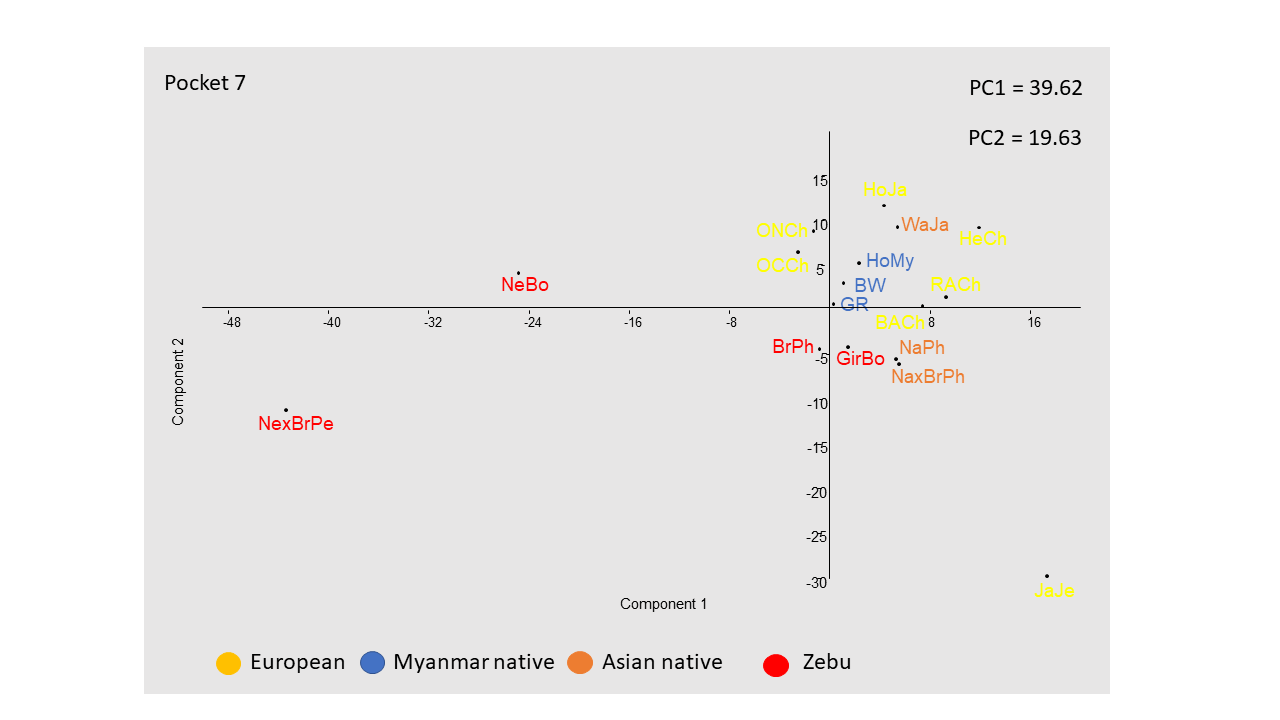


e.


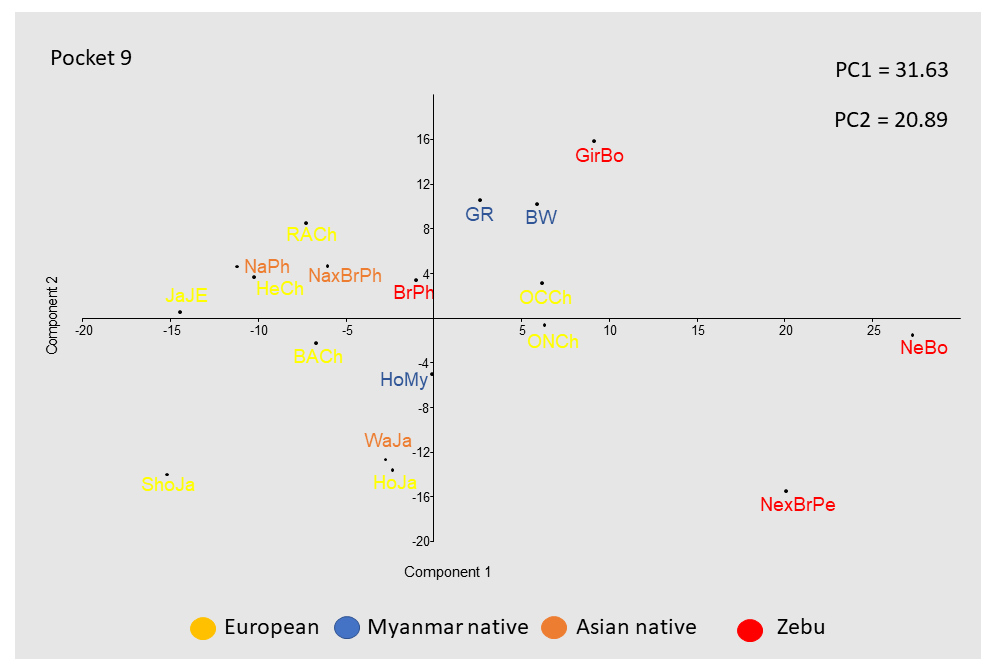

Supplement: Supplementary file 2 — Additional file 2: Figure S2. Principal component analysis of BoLa-DRB3 gene pocket amino acid motifs frequencies in 18 populations: a. Pocket 1, b. Pocket 4, c. Pocket 6, d. Pocket 7, and e. Pocket 9. BW = Pyer Sein, GR = Shwe Ni, HoMy = Myanmar Holstein-Friesian crossbreed, WaJa = Japanese Black, HoJa = Holstein, ShJa = Japanese Shorthorn, JeJa = Jersey, HeCh = Chilean Hereford, BACh = Chilean Black Angus, RACh = Chilean Red Angus, ONCh = Chilean Overo Negro, OCCh = Chilean Overo Colorado, NaPh = Philippine Native, NaxBrPh = Native x Brahman Philippine crossbreed, BrPh = Philippine Brahman, NeBo = Bolivian Nellore, GirBo = Bolivian Gir, and BrxNe = Peruvian Brahman × Nellore crossbreed. [file 12863_2020_905_MOESM2_ESM.docx]
