# Supplementary material for: Characterization of bovine MHC DRB3 diversity in global cattle breeds, with a focus on cattle in Myanmar
Source: BMC Genet. 2020 Sep 1;21:95. doi: 10.1186/s12863-020-00905-8 (PMC7460757; doi:10.1186/s12863-020-00905-8)
Supplement: Supplementary file 3 — Additional file 3: Figure S3. Geographical distribution of sampling sites of Myanmar cattle that are indicated by a dot. [file 12863_2020_905_MOESM3_ESM.docx]

**Fig. S3.** Geographical distribution of sampling sites of Myanmar cattle that are indicated by a dot.


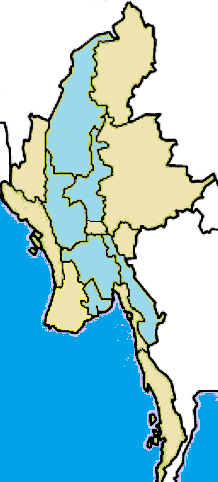


**Bago Region**

**(N = 38)**

Thailand

India

China

**Sagaing Region**

**(N = 77)**

**Magway Region**

**(N = 46)**

**Yangon Region**

**(N = 44)**

**Kayin State**

**(N = 43)**

**Mandalay Region**

**(N = 46)**

Laos

Indian Ocean
