# Supplementary material for: Characterization of bovine MHC DRB3 diversity in global cattle breeds, with a focus on cattle in Myanmar
Source: BMC Genet. 2020 Sep 1;21:95. doi: 10.1186/s12863-020-00905-8 (PMC7460757; doi:10.1186/s12863-020-00905-8)
Supplement: Supplementary file 4 — Additional file 4: Table S1. Detailed information about the populations analyzed. [file 12863_2020_905_MOESM4_ESM.docx]

**Table S1** Detailed information about the populations analyzed

| Acronym | Sample size | Breed | Type | Origin (country) | Sampling country | Reference |
| --- | --- | --- | --- | --- | --- | --- |
| BW | 163 | Pyersein | Zebu | Myanmar | Myanmar | Present work |
| GR | 69 | Shweni | Zebu | Myanmar | Myanmar | Present work |
| HoMy | 62 | Holstein x Friesian | taurine | Netherlands | Myanmar | Present work |
| HV | 66 | Hartón del Valle | taurine | Colombia | Colombia | Giovambattista et al., 2013 |
| YA | 112 | Yacumeño Creole | taurine | Bolivia | Bolivia | Giovambattista et al., 2013 |
| NeBo | 116 | Nellore | Zebu | Brazil | Bolivia | Takeshima et al., 2018 |
| GirBo | 110 | Gir | Zebu | India | Bolivia | Takeshima et al., 2018 |
| NexBrPe | 195 | Nellore × Brahman | Zebu mixed | - | Peru | Takeshima et al., 2018 |
| HoJa | 102 | Japanese Holstein | taurine | Netherlands | Japan | Takeshima et al., 2003 |
| ShJa | 100 | Japanese Shorthorn | taurine | Great Britain | Japan | Takeshima et al., 2003 |
| JeJa | 69 | Japanese Jersey | taurine | Channel Island | Japan | Takeshima et al., 2003 |
| WaJa | 200 | Japanese Black | taurine | Japan | Japan | Takeshima et al., 2003 |
| HeCh | 49 | Hereford | taurine | Great Britain | Chile | Takeshima et al., 2015 |
| BACh | 100 | Chilean Black Angus | taurine | Great Britain | Chile | Takeshima et al., 2015 |
| RACh | 99 | Chilean Red Angus | taurine | Great Britain | Chile | Takeshima et al., 2015 |
| ONCh | 124 | Chilean Overo Negro | taurine |  | Chile | Takeshima et al., 2015 |
| OCCh | 136 | Chilean Overo Colorado | taurine |  | Chile | Takeshima et al., 2015 |
| BrPh | 236 | Brahman | Zebu | USA | Philippines | Polat et al., 2014 |
| NaPh | 482 | Philippine Native | taurine | Philippines | Philippines | Polat et al., 2014 |
| NaxBrPh | 132 | Native x Brahman | Zebu mixed | - | Philippines | Polat et al., 2014 |
