# Supplementary material for: Characterization of bovine MHC DRB3 diversity in global cattle breeds, with a focus on cattle in Myanmar
Source: BMC Genet. 2020 Sep 1;21:95. doi: 10.1186/s12863-020-00905-8 (PMC7460757; doi:10.1186/s12863-020-00905-8)
Supplement: Supplementary file 5 — Additional file 5: Table S2. Genetic distance between pairs of populations estimated by FST in (a) six Myanmar native (KN = Kayin, BN = Bago, SN = Sagaing, MdN = Mandalay, MgN = Magway, and YN = Yangon) and (b) four Myanmar Holstein-Friesian crossbreed (KF = Kayin, BF = Bago, SF = Sagaing, and YF = Yangon) populations. [file 12863_2020_905_MOESM5_ESM.docx]

**Table S2**. Genetic distance between pair of populations estimated through F_ST_ (above) and Nei D_A_ distance (below). Pyer Sein (BW). Shwe Ni (GR), Myanmar Holstein-Friesian crossbreed (HoMy), Bolivian Nellore (NeBo), Bolivian Gir (GirBo), Peruvian Brahman × Nellore crossbreed (BrxNe), Japanese Holstein (HoJa), Japanese Shorthorn (ShJa), Japanese Jersey (JeJa), Japanese Black (WaJa), Chilean Hereford (HeCh), Chilean Black Angus (BACh), Chilean Red Angus (RACh), Chilean Overo Negro(ONCh), Chilean Overo Colorado (OCCh), Philippine native (NaPh), and Philippine Brahman (BrPh).
