# Supplementary material for: Characterization of bovine MHC DRB3 diversity in global cattle breeds, with a focus on cattle in Myanmar
Source: BMC Genet. 2020 Sep 1;21:95. doi: 10.1186/s12863-020-00905-8 (PMC7460757; doi:10.1186/s12863-020-00905-8)
Supplement: Supplementary file 6 — Additional file 6: Table S3. Genetic distance between pairs of populations estimated by FST in (a) six Myanmar native (KN = Kayin, BN = Bago, SN = Sagaing, MdN = Mandalay, MgN = Magway, and YN = Yangon), and (b) four Myanmar Holstein-Friesian crossbreed (KF = Kayin, BF = Bago, SF = Sagaing, and YF = Yangon) populations. [file 12863_2020_905_MOESM6_ESM.docx]

**Table S3.** Genetic distance between pairs of populations estimated by F_ST_ in (a) six Myanmar native (KN = Kayin, BN = Bago, SN = Sagaing, MdN = Mandalay, MgN = Magway, and YN = Yangon), and (b) four Myanmar Holstein-Friesian crossbreed (KF = Kayin, BF = Bago, SF = Sagaing, and YF = Yangon) populations.

a)

|  | BN | KN | MdN | MgN | SN | YN |
| --- | --- | --- | --- | --- | --- | --- |
| BN | 0 |  |  |  |  |  |
| KN | 0.007 | 0 |  |  |  |  |
| MdN | 0.023 | 0.022 | 0 |  |  |  |
| MgN | 0.004 | **0.007** | 0.016 | 0 |  |  |
| SN | **0.008** | **0.004** | **0.024** | **0.011** | 0 |  |
| YN | **0.009** | **0.009** | **0.008** | 0.003 | **0.009** | 0 |

b)

|  | BF | KF | SF | YF |
| --- | --- | --- | --- | --- |
| BF | 0 |  |  |  |
| KF | 0.014 | 0 |  |  |
| SF | 0.003 | **0.031** | 0 |  |
| YF | 0 | 0.015 | 0.011 | 0 |
